# Prevalence and Control of *Pseudomonas aeruginosa* in Tourist Facilities across the Canary Islands, Spain

**DOI:** 10.3390/pathogens13060501

**Published:** 2024-06-12

**Authors:** Antonio Doménech-Sánchez, Elena Laso, Sebastián Albertí

**Affiliations:** 1 Instituto Universitario de Investigación en Ciencias de la Salud (IUNICS), Universidad de las Islas Baleares, Carretera de Valldemossa km 7.5, 07122 Palma de Mallorca, Spain; sebastian.alberti@uib.es; 2 Saniconsult Ibérica SL, Can Foradí 37 bajos, 07009 Palma de Mallorca, Spain; laso.mariaelena@gmail.com; 3 Instituto de Investigación Sanitaria de les Illes Balears (IdIsBa), Edificio S, Hospital Universitario Son Espases, Carretera de Valldemossa 79, 07120 Palma de Mallorca, Spain

**Keywords:** hotel, chlorine, bromine, swimming pool, children, disinfectants

## Abstract

*Pseudomonas aeruginosa* is a common pathogen associated with recreational water facilities and poses risks to public health. However, data on the prevalence of *P. aeruginosa* in tourist destinations like the Canary Islands, Spain, remain limited. We assessed *P. aeruginosa* prevalence in 23 tourist facilities from 2016 to 2019. Compliance with water quality standards was evaluated, and 3962 samples were collected and analyzed. We examined different types of recreational water installations, including outer swimming pools, whirlpools, and cold wells. Of the sampled facilities, 31.2% did not comply with the current legislation’s parametric values, mainly due to inadequate disinfectant levels, water temperature, and *P. aeruginosa* presence. The prevalence of *P. aeruginosa* was 4.8%, comparable to some European countries but lower than others. Cold wells displayed the highest non-compliance rate (89.2%) and yet exhibited a lower *P. aeruginosa* prevalence (1.9%) than outer swimming pools and whirlpools. Children’s presence did not significantly impact *P. aeruginosa* contamination. Chlorine-based disinfectants are more effective than bromine-based ones in controlling *P. aeruginosa*. Regional variability in contamination was observed, with Fuerteventura showing lower colonization rates. Disinfectant levels play a critical role in *P. aeruginosa* control, and maintaining adequate levels is essential, particularly in bromine-treated installations. Our findings provide valuable insights into the prevalence and distribution of *P. aeruginosa* in recreational waters within tourist facilities. Tailored strategies are needed to ensure water safety in different Spanish regions. Continued monitoring and assessment, combined with artificial intelligence and machine learning, will enable the implementation of targeted interventions to protect the health of recreational water users.

## 1. Introduction

*Pseudomonas aeruginosa* is an exceptionally versatile bacterium, capable of thriving in diverse environments, including inert surfaces and living organisms such as plants, animals, and humans [1]. This microorganism is notorious for causing various human pathologies, often affecting the skin, ears, eyes, as well as the respiratory and urinary tracts. Particularly concerning is its impact on immunocompromised individuals, leading to severe respiratory and bloodstream infections with high morbidity and mortality rates [2]. Adding to the challenge is the frequent occurrence of therapeutic failures due to the development of antimicrobial resistance in this pathogen. In Europe, a considerable percentage (30.1%) of *P. aeruginosa* isolates exhibited resistance to at least one antimicrobial group under surveillance, with 9.4% of isolates showing resistance to three or more antimicrobials [3]. The emergence of antibiotic-resistant infections has also incurred substantial additional hospital care costs, estimated at around EUR 1.6 billion in 2012 [4]. Consequently, the World Health Organization has classified multidrug-resistant *P. aeruginosa* as a “Priority 1 Critical Pathogen” for the research and development of new antibiotics [5].

Recreational water facilities, including swimming pools, whirlpools, and aquatic parks, play a significant role in promoting well-being, fitness, leisure, and health. Unfortunately, these amenities are sometimes associated with infections caused by various microorganisms, particularly *P. aeruginosa*. Swimmers may experience otitis externa, folliculitis, dermatitis, hot-foot syndrome, ocular infections, and, in more severe cases, pneumonia and urinary infections [6,7,8,9,10]. To ensure the health and safety of users, the proper maintenance of these installations, including filtration and disinfection processes, is vital. However, establishing consistent standards for well-managed swimming pools is challenging due to varying regulations among different countries [11]. In Spain, until 2013, local authorities dictated separate legislation and guidelines, which led to discrepancies in water quality criteria. Subsequently, the Spanish government established a guide to standardize criteria nationwide [12].

The Canary Islands stand as one of Spain’s primary tourist destinations [13], where ensuring safe and healthy conditions is of utmost importance. While many tourists enjoy the beaches, hotels in the region often offer swimming pools to meet the high demand from visitors and tour operators. Hotel managers face substantial fines for each day their swimming pools remain unavailable, making impeccable pool maintenance essential to avoid health-related issues and associated losses from closure. Several studies have examined the microbiological conditions of swimming pools in different countries; however, limited information is available concerning Spanish pools, specifically concerning *P. aeruginosa*. Previous investigations into the presence of *P. aeruginosa* in Spain were conducted with constraints on sample size and study period, and they were performed before the implementation of current legislation [14]. Therefore, comprehensive and up-to-date research is required to understand the current state of recreational waters in our country, especially in the context of the tourist sector.

This study aimed to survey recreational water installations in hotels across the Canary Islands, Spain, to assess compliance with current standards, investigate the prevalence of *Pseudomonas aeruginosa*, and explore its relationship with various physicochemical and microbiological parameters over four years. This knowledge will enable the development of specific measures to mitigate the *P. aeruginosa* infection risk associated with these amenities.

## 2. Materials and Methods

### 2.1. Study Facilities

This investigation encompassed a total of 23 distinct tourist accommodations in the Canary Islands, Spain, comprising hotels and apartments. The visits were conducted between 9 January 2016, and 21 November 2019, with the aim of obtaining an up-to-date understanding of the prevailing conditions. To ensure an accurate representation, the management of these facilities was not notified in advance, and samples were collected under normal operational conditions. On average, each installation was visited 43 times. The study focused on analyzing recreational water in various facilities, including swimming pools, cold wells, whirlpools, hot tubs, and other warm water accommodations associated with spas. For simplicity, we will refer to these facilities collectively as whirlpools throughout the rest of this manuscript.

### 2.2. Sampling Procedure

Sampling points were strategically determined based on worst-case scenarios, taking into consideration pool characteristics such as shape, depth, and the positioning of injectors, skimmers, and drains, among others. A single sampling point was designated for each swimming pool, where water samples were collected at a depth of 30 cm. Sterile plastic bottles were used to collect water samples, and the disinfectant present in the water was neutralized using sodium thiosulfate directly in the sampling container (Sharlab, Barcelona, Spain). The samples were promptly placed in a portable refrigerator and transported to the laboratory.

Several parameters were measured on site, including temperature, pH, and the levels of disinfectant and cyanuric acid. Temperature measurements were taken using a Testo 104 thermometer (Testo, Barcelona, Spain) in water-temperature-controlled facilities such as indoor swimming pools, whirlpools, and cold wells. Disinfectant levels (both free and combined chlorine or bromine), as well as cyanuric acid levels, were measured using a Lovibond^®^ portable MD100 instrument (Lovibond, Dortmund, Germany), following previously established methods [15]. The same instrument was employed for pH determination using a colorimetric method [16].

### 2.3. Laboratory Investigation

Upon arrival at the laboratory, the water samples were subjected to analysis for turbidity, conductivity, and total dissolved solids (TDS) using methods previously described [16]. Microbiological analysis involved the detection and enumeration of *Escherichia coli* and *Pseudomonas aeruginosa* in 100 mL of water. For both microorganisms, detection was based on bacterial enzyme detection technology that indicates the presence of the target microorganism. Colilert-18^®^ was used to detect *E. coli* in water (ISO 9308-2:2012) [17], while Pseudalert^®^ served as the standard method for *P. aeruginosa* detection (ISO: 16266-2:2018) [18].

### 2.4. Data Analysis

Data from the QUAASS-LAB^®^ LIMS system were carefully reviewed to identify and address any duplicate or inconsistent entries, resulting in a final dataset comprising 3962 samples. The D’Agostino–Pearson normality test was applied to assess the normal distribution of quantitative parameters, followed by the Mann–Whitney test for non-normally distributed data. Fisher’s exact test was employed for analyzing qualitative parameters. Correlation analysis was conducted using Spearman’s rank correlation coefficient (r). Statistical significance was determined when the *p*-value was less than 0.05. All statistical analyses were performed using GraphPad Prism software version 9.4.1.

## 3. Results

### 3.1. Compliance of Tourist Swimming Pools with Water Standards

**Figure 1:**
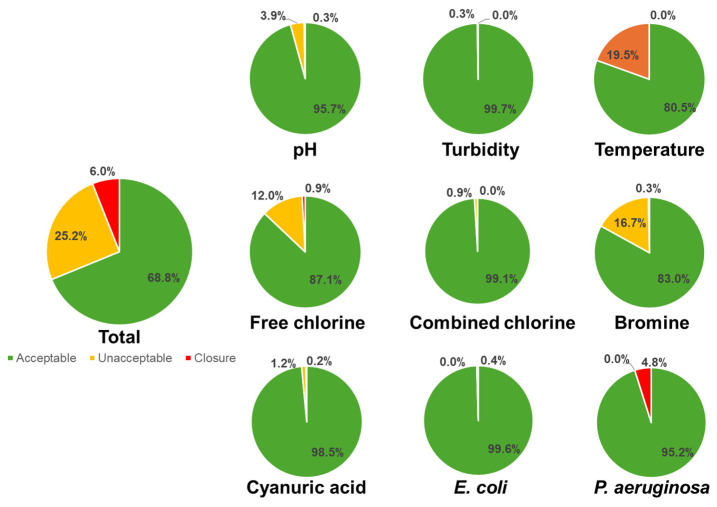
Compliance with water standards of legislated parameters in the investigated pools from hotels in the Canary Islands.

The study encompassed a comprehensive analysis of 3962 samples collected from 23 tourist facilities (mean 172 samples/hotel; median 173 samples/hotel) across the Canary Islands over four years (2016–2019). Figure 1 presents the results based on the parametric values mandated by the existing legislation [12], categorizing samples as either acceptable or unacceptable. Approximately 31.2% of the samples (1235 out of 3962) exhibited unacceptable levels of at least one parameter. The parameters most frequently deviating from established limits were disinfectants (free chlorine and bromine) and water temperature. While values exceeding the parametric limits do not necessarily imply immediate health hazards for users, the legislation specifically requires closure of installations in cases where certain thresholds are exceeded, such as temperature > 40 °C, pH < 6.0 or >9.0, turbidity > 20 NFU, free chlorine > 5 mg L^−1^, combined chlorine > 3 mg L^−1^, cyanuric acid > 150 mg L^−1^, and bromine 10 mg L^−1^. Notably, *P. aeruginosa* emerged as a significant parameter responsible for pool closures, particularly in cases where the mandatory microbiological levels were not met. Based on these levels, 6.0% of the sampled installations should have been closed. The data in Figure 1 underscore the significant role of *P. aeruginosa* as the primary cause of non-compliance leading to pool closures, with considerable variations observed among different types of installations. Cold wells displayed the highest non-compliance rate (89.2% of samples), followed by outer swimming pools (25.6%) and whirlpools (20.6%). In contrast, the three samples collected from inner swimming pools showed 100% non-compliance.

### 3.2. P. aeruginosa Contamination in Different Types of Recreational Water Installations

**Figure 2:**
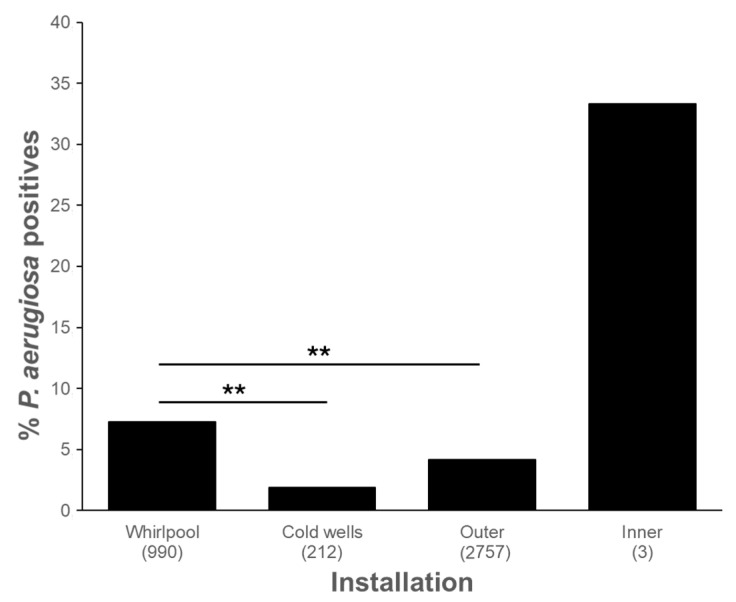
Contamination of *P. aeruginosa* in the different types of investigated pools in hotels located in the Canary Islands. The number of the analyzed samples is shown in brackets (**, *p* < 0.01).

Tourist facilities offer various recreational water installations, classified into four groups in this study: outer and inner swimming pools, whirlpools, and cold wells. Figure 2 displays the results of *P. aeruginosa* contamination in each group. Overall, the pathogen was detected in 4.8% of the samples. Notably, whirlpool samples exhibited significantly higher contamination rates (7.3%) compared to outer swimming pools and cold wells (4.2% and 1.9%, respectively; *p* < 0.01). However, no significant differences were found for inner swimming pools due to the limited number of samples available for analysis.

**Figure 3:**
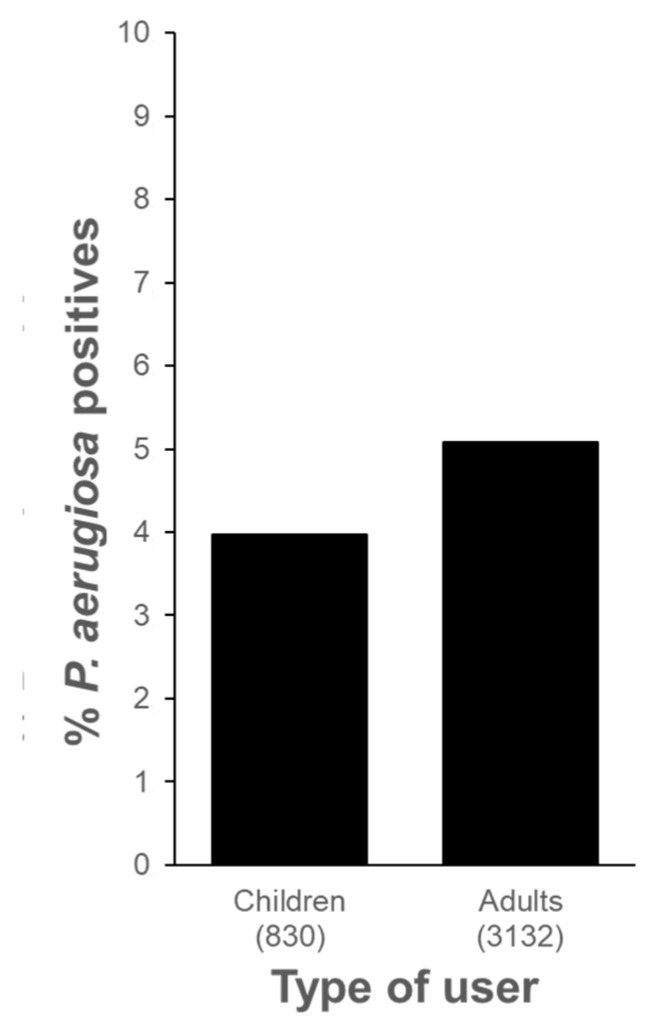
Contamination of *P. aeruginosa* in pool water samples from adults’ and children’s pools in hotels located in the Canary Islands. The number of the analyzed samples is shown in brackets.

Additionally, the study compared contamination rates between pools used by children and those used by adults (Figure 3). The percentages of contaminated samples were found to be very similar in both groups (4.0% and 5.1%, respectively), with no statistically significant differences observed between the two (*p* = 0.231).

### 3.3. Relationship between P. aeruginosa Presence and Water Disinfection Methods

**Figure 4:**
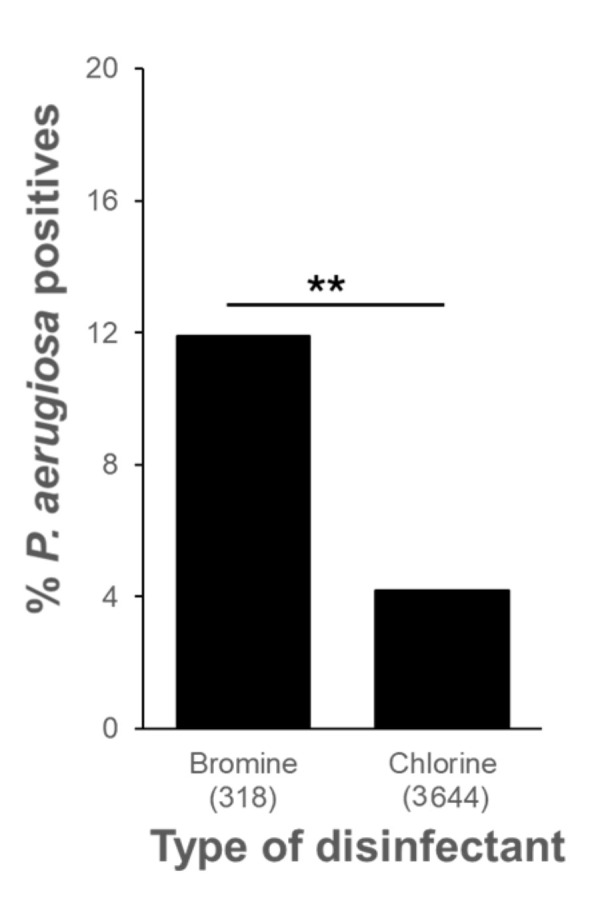
Contamination of *P. aeruginosa* in pool water samples with different disinfectant treatments in hotels located in the Canary Islands. The number of the analyzed samples is shown in brackets (**, *p* < 0.01).

Water treatment in pool basins often involves various options, with chlorine being the most used method in the region [16]. However, the survey also included facilities employing bromine for disinfection. The comparison between recreational waters treated with chlorine and bromine is presented in Figure 4. The results indicate that *P. aeruginosa* contamination is significantly influenced by the disinfection method used. Specifically, the percentage of contamination in bromine-treated samples (11.9%) was nearly three times higher than that observed in chlorine-treated ones (4.2%).

**Figure 5:**
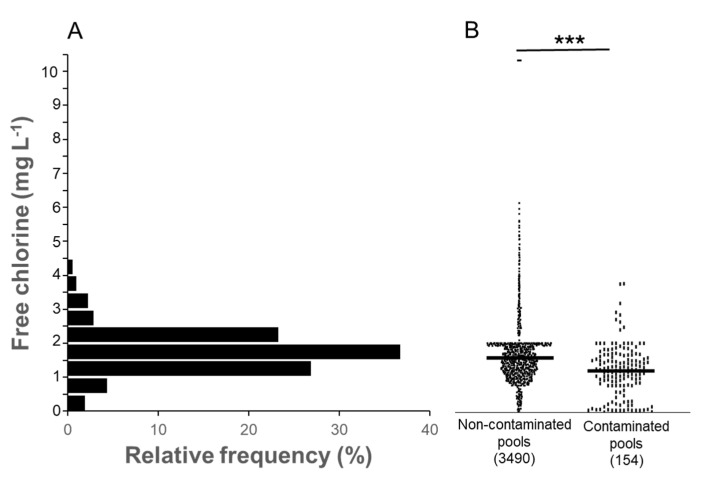
Frequency distribution of free chlorine levels in *P. aeruginosa*-contaminated and not contaminated pool samples in the Canary Islands tourist resorts. (**A**) Histogram showing the relative frequency of free chlorine levels in ranges of 0.5 mg L^−1^. (**B**) Comparison of free chlorine levels in contaminated and non-contaminated pools. The number of the analyzed samples is shown in brackets (***, *p* < 0.001).

**Figure 6:**
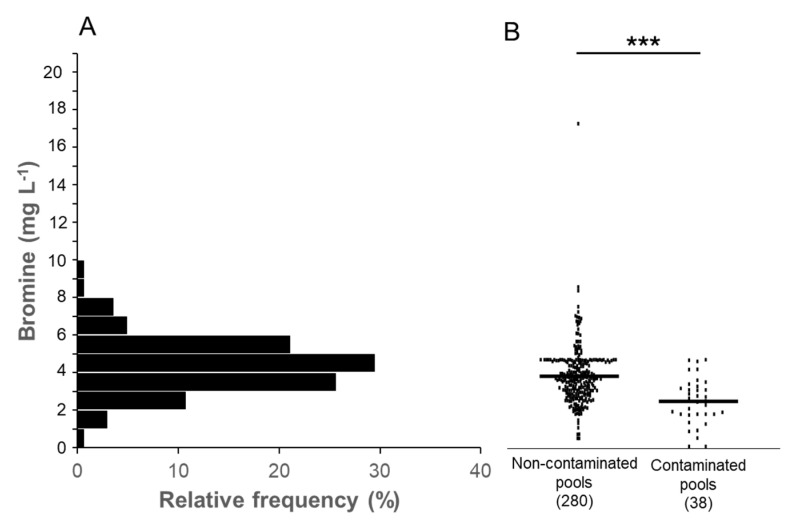
Frequency distribution of bromine levels in *P. aeruginosa*-contaminated and not contaminated pool samples in Canary Islands tourist resorts. (**A**) Histogram showing the relative frequency of bromine levels in ranges of 1 mg L^−1^. (**B**) Comparison of bromine levels in contaminated and non-contaminated pools. The number of the analyzed samples is shown in brackets (***, *p* < 0.001).

Furthermore, an individual analysis of disinfectant levels revealed that free chlorine concentration was predominantly between 1 and 2.5 mg L^−1^ (Figure 5A). Notably, contaminated samples exhibited significantly lower free chlorine levels (*p* < 0.001, Figure 5B). In the case of bromine, the concentration was mainly between 3 and 6 mg L^−1^ (Figure 6A), with a similar observation of statistically significant differences in the presence or absence of the pathogen in water samples (*p* < 0.001, Figure 6B).

### 3.4. Geographical Distribution of P. aeruginosa Contamination in Tourist Facilities

The water samples collected in this study originated from four different islands within the Canary Islands archipelago: Fuerteventura, Gran Canaria, Lanzarote, and Tenerife. Positive samples were obtained from all locations, with the colonization rate varying across the islands: 2.9% in Fuerteventura, 4.9% in Gran Canaria, 5.2% in Lanzarote, and 5.4% in Tenerife. Statistical analysis indicated that contamination levels in Fuerteventura were significantly lower than those observed in the other islands (*p* < 0.05). However, no significant differences were observed in disinfectant levels among the different islands.

### 3.5. Evolution of P. aeruginosa Contamination over Time

**Figure 7:**
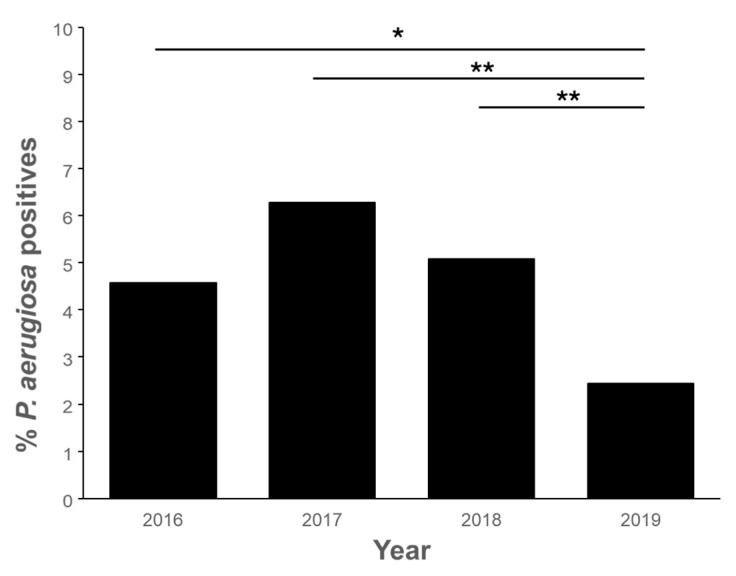
Progress of the contamination of *P. aeruginosa* over the years in pools located in the Canary Islands (*, *p* < 0.05; **, *p* < 0.01).

The longitudinal nature of the investigation spanning four years allowed the assessment of changes in *P. aeruginosa* contamination rates over different periods. Figure 7 presents the evolution of pathogen contamination over the years, showing relatively stable contamination levels, around 4–6% from 2016 to 2018. However, in 2019, a notable decrease in *P. aeruginosa* contamination was observed, with the rate dropping below 2.5%.

**Figure 8:**
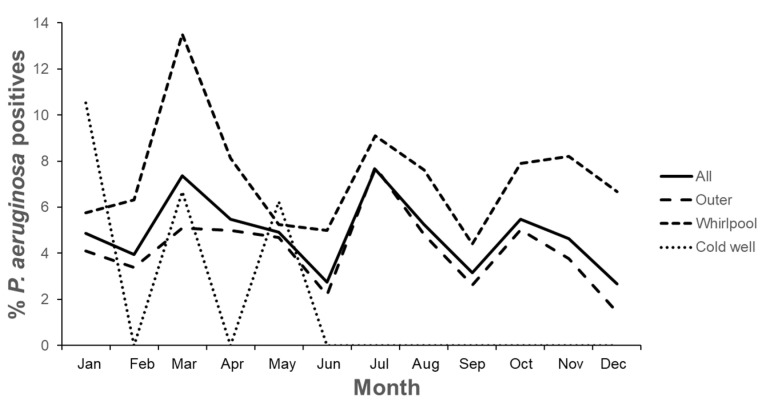
Seasonal frequency of *P. aeruginosa* contamination in tourist pools located in the Canary Islands. Inner pools are not included because of the limited number of samples.

Additionally, the study monitored seasonal variations monthly, as depicted in Figure 8, which provides a graphic showing the frequency of sample contamination by month and installation. Notably, indoor pool values are not displayed to avoid distortions due to the limited number of samples. *P. aeruginosa* was most frequently present in recreational waters in March and July, with a decrease in pathogen presence in June and December. Some deviations from this pattern were observed when analyzing specific installations, such as high pathogen levels in cold wells detected in January.

### 3.6. Effects of Water Parameters on P. aeruginosa Presence 

The study included the measurement of several parameters to monitor the sanitary conditions of the pools, facilitating the investigation of their effects on *P. aeruginosa* presence. Table 1 presents the main parameters analyzed for correlation with *P. aeruginosa.* A significant correlation (*p* < 0.05) was observed between *P. aeruginosa* and *E. coli* (rs = 0.12) and temperature (rs = 0.07) and turbidity (rs = 0.06). Conversely, a negative correlation was found between P. aeruginosa and bromine (rs= −0.30) as well as free chlorine (rs = −0.10). No significant correlations were observed between *P. aeruginosa* and pH, combined chlorine, cyanuric acid, conductivity, and TDS. However, the Spearman correlation coefficient values were consistently low, indicating minimal or no correlation between water characteristics and *P. aeruginosa* contamination.

## 4. Discussion

Spain stands as one of the prime European tourist destinations, boasting a well-established and thriving hotel sector. Recreational water services, including swimming pools and similar facilities, are in high demand among visitors. *Pseudomonas aeruginosa* infections have been extensively reported in association with the use of these recreational waters, making it one of the most linked pathogens [9,10]. However, information on the prevalence of this bacterium in recreational water facilities in Spain, particularly in tourist destinations, remains scarce. This study aimed to investigate the prevalence of *P. aeruginosa* in hotels situated in the Canary Islands, a major Spanish tourist destination.

First, we assessed the degree of compliance with water standards in the visited recreational water installations. The study collected and analyzed 3962 samples from 23 tourist facilities over four years (2016–2019). The results revealed that 31.2% of the samples did not meet the current legislation’s parametric values, with disinfectant levels, water temperature, and the presence of *P. aeruginosa* being the most common reasons for non-compliance (Figure 1). Among the examined parameters, 6.0% of the sampled installations should have been closed due to the presence of pathogenic levels, with *P. aeruginosa* being the primary cause. Cold wells displayed the highest incidence of non-compliance (89.2%), followed by outer swimming pools (25.6%) and whirlpools (20.6%), while the number of inner swimming pool samples was insufficient for statistical analysis. The non-compliance rate in Spanish facilities was higher than the 19.1% reported in Bologna, Italy [19], where outer swimming pools showed similar figures (25%). Moreover, the non-conformity of samples led to pool closure only in 1.5% of cases, in contrast to our 6.0%. However, in the same country, pools in Milan had higher incompliance rates, reaching 72.3% in some cases [20]. Comparisons among countries are challenging due to the differences in standards [21], which underscores the importance of individual investigations.

Next, our attention turned to the prevalence of *P. aeruginosa*, and the results revealed a prevalence of 4.8% (Figure 2). This finding places the prevalence within the range reported in some European countries, while comparatively lower than others. For instance, studies from Croatia and Italy reported similar prevalence rates [19,20], whereas those in Greece [22,23] and Northern Ireland [21] were clearly higher. Higher levels of *P. aeruginosa* contamination were also present in non-European countries, like Australia, Egypt, and Brazil [24,25,26]. However, it is essential to consider regional and climatic variations, as they can significantly impact *P. aeruginosa* colonization in recreational waters. An intriguing observation from our investigation was the variation in *P. aeruginosa* contamination among different types of recreational water facilities. Cold well pools, despite showing higher non-compliance rates (see Section 3.1), exhibited a lower prevalence of *P. aeruginosa* (1.9%) compared to outer swimming pools and whirlpools. This counterintuitive finding may be attributed to the role of water temperature in affecting *Pseudomonas* growth. Cold wells’ lower water temperature, typically below 15 °C, might create less favorable conditions for *P. aeruginosa* proliferation, despite the increased likelihood of other contaminants in these pools. While the prevalence of *P. aeruginosa* contamination in whirlpools (7.3%) was slightly higher than in outer swimming pools (4.2%), the differences between the two were not statistically significant, consistent with recent findings in Croatia [27]. Conversely, a study by Tirodimos et al. reported lower contamination rates in whirlpools compared to outer swimming pools [22]. Our investigation revealed that the rate of *Pseudomonas* contamination in whirlpools was comparable to that observed in whirlpools from Greece and Croatia [22,27], indicating some consistency in the prevalence of the pathogen across different regions. However, whirlpools in Northern Ireland exhibited more severe sanitary conditions, reporting higher levels of *Pseudomonas* contamination [21]. These variations between countries may be influenced by differences in water treatment practices, pool maintenance, and user behavior, underscoring the importance of regional context in assessing recreational water safety.

Additionally, our study delved into the influence of child users on *P. aeruginosa* contamination in recreational waters. Interestingly, we found no significant impact, indicating that both children’s and adults’ pools pose similar risks in terms of *P. aeruginosa* prevalence (Figure 3). While previous studies have highlighted the importance of maintaining proper water hygiene in children’s pools, our findings suggest that additional factors may contribute to pathogen presence, beyond the age of the pool users.

Maintaining safe conditions for swimming pool users and preventing microbial contamination is crucial in recreational water management. This objective is achieved by employing disinfectants with potent bactericidal effects to control microorganisms present in the water. While previous surveillance studies on *P. aeruginosa* in swimming pools predominantly focused on chlorine-based disinfectant treatments, our investigation included both chlorine and bromine treatments. Our findings revealed that chlorine exhibited greater efficacy than bromine, as the presence of the pathogen was three times higher in the latter compared to the former. Chlorine is widely regarded by most health authorities as the preferred disinfectant for swimming pool water due to its reliable performance in maintaining water quality and reducing pathogen levels. It is noteworthy that our study is the first of its kind to evaluate the use of bromine-based disinfectant treatments in swimming pool surveys. This exploration of bromine’s effectiveness contributes valuable insights to the field of recreational water safety. Additionally, we conducted a comparative analysis of disinfectant concentrations in both contaminated and non-contaminated samples. As anticipated, the disinfectant levels were significantly lower (*p* < 0.001) in the contaminated samples, regardless of whether chlorine or bromine was used for treatment. This underscores the importance of maintaining appropriate disinfectant levels in the water to effectively control microbial contamination and ensure the safety of pool users.

Regarding the geographical distribution of *P. aeruginosa* contamination in the Canary Islands, our analysis indicated a significantly lower colonization rate in Fuerteventura compared to the other islands. Curiously, a similar trend was observed for *Legionella*, another aquatic pathogen associated with recreational waters [28]. These findings highlight the importance of considering regional variability in the prevalence and distribution of pathogens, which can be influenced by local environmental conditions and specific management practices. As a result, tailored and region-specific approaches to water safety and management become essential to ensure the well-being of recreational water users in tourist destinations like the Canary Islands.

The scope of our investigation extended over a period of four years, allowing us to analyze the temporal evolution of *P. aeruginosa* contamination (Figure 7). The prevalence of the pathogen remained relatively stable throughout the study, ranging between 4% and 6% in the earlier years. However, a significant decline in contamination, below 2.5%, was observed in 2019. Interestingly, a contrasting trend was reported in Croatian pools during the same period [27]. In that study, the increase in *P. aeruginosa* contamination was attributed to a record surge in tourist visits in 2019. This suggests that the number of tourists visiting the Canary Islands may also play a role in influencing *Pseudomonas* prevalence in our case. Indeed, when *Pseudomonas* prevalence was below 5% (years 2016 and 2019), the Canary Islands received 13 million tourists, while during years with prevalence above 5% (2017 and 2018), the number of visits increased to 14 million [13]. Despite this influence, it is crucial to consider the effectiveness of disinfection practices over the years. However, our analysis revealed that disinfectant levels remained similar over time, dismissing this factor as a direct cause for the changes in contamination rates. Subsequently, we explored monthly variations in *P. aeruginosa* presence (Figure 8). Unlike the seasonal differences described in the Croatian study [27], where *P. aeruginosa* prevalence decreased during the winter and spring seasons, our study in the Canary Islands did not show significant seasonal variations. This discrepancy could be attributed to the stable temperatures characteristic of subtropical climates, which contrast with the more pronounced seasonal changes in Mediterranean regions. However, distinct patterns emerged when comparing data from different types of installations. Notably, the contamination of cold wells exhibited a notable increase in January, contrasting with other pool types.

Lastly, we conducted a comprehensive examination of the associations between *P. aeruginosa* contamination and various physicochemical and microbiological parameters (Table 1). Utilizing the Spearman correlation coefficient, our analysis revealed predominantly weak or no correlations between the presence of the pathogen and most parameters. However, we observed a consistent positive correlation between *P. aeruginosa* and *E. coli* levels, which is in line with findings from previous studies [24,27,29,30], underscoring that both fecal and non-fecal shedding contribute to *P. aeruginosa* contamination in pool water and the surrounding environment. Additionally, we found weak positive correlations between the pathogen and turbidity and temperature, two factors that have previously been linked to microbial contamination in water [31]. On the other hand, we observed a very weak negative correlation between *P. aeruginosa* and disinfectant levels, which aligns with previous investigations [24,26,27], reinforcing the critical importance of maintaining appropriate disinfectant levels, particularly in bromine-treated installations, to ensure effective water sanitation. Notably, we did not find any significant correlation between *P. aeruginosa* and pH levels in our analysis, a parameter that has shown varying correlations in different studies [24,26].

The management and control of swimming pools require the monitoring of numerous parameters that fluctuate over time due to various factors, such as different treatments applied, environmental conditions, and the presence of users, among others. Generally, the most critical parameters, such as pH and disinfectant levels, are manually controlled by maintenance personnel, who measure them using field kits. Other parameters are monitored much less frequently, typically by external consultants and laboratories. An improvement in this regard has been the implementation of automatic sensors that continuously measure pH and disinfectant levels in the water, injecting the necessary products based on the results obtained. However, the next step would be applying automated systems based on artificial intelligence. Their application provides technical support to the operational processes of water control, which is much more efficient than relying solely on human operations. These data analyses based on AI and machine learning will enable diagnostics, automatic decision-making, and the optimization of operational processes [32,33].

Numerous factors could potentially explain the differences between our study and previously published surveys, including variations in national standards (where available), differences in the number of pools and samples investigated, selection of specific parameters, varying pool maintenance practices, and the unique characteristics of the installations and user demographics. Our study stands out as one of the most comprehensive, involving a substantial number of samples and encompassing different types of recreational waters while incorporating most of the legislated parameters. Furthermore, our investigation delved into the impact of bromine treatment and child users on *P. aeruginosa* contamination in recreational waters, providing valuable insights into these less-explored aspects. As such, our results provide a highly consistent representation of the prevalence of *P. aeruginosa* colonization in swimming pools within tourist facilities, at least within our country. Nevertheless, certain limitations must be acknowledged in our study. The number of samples from inner swimming pools is relatively low, primarily due to the favorable warm temperatures in the Canary Islands, allowing the predominant use of outdoor pools throughout the year. Additionally, *Legionella* spp., a parameter also endorsed by legislation, was not included in our analysis; however, a specific and extensive survey on this pathogen in the region was recently published [28]. It is essential to consider that our study concluded before the COVID-19 pandemic, and current practices and regulations may have evolved in response to the new preventive measures.

In conclusion, our investigation provided an in-depth characterization of P. aeruginosa prevalence in tourist recreational water installations in the Canary Islands, Spain. Our extensive analysis of various facilities revealed varying degrees of contamination. We highlight the significance of maintaining appropriate disinfectant levels, especially in bromine-treated installations, to ensure water safety. Nonetheless, further studies in different regions across Spain are warranted, as regional variability in water quality and contamination has been observed. By continuing to monitor and assess recreational water quality, tailored strategies and targeted interventions can be implemented to safeguard the health and well-being of recreational water users in tourist destinations like the Canary Islands.

## Figures and Tables

**Table 1:** Correlation analysis of pool water characteristics associated with *P. aeruginosa* contamination in the Canary Islands.

Parameter	Spearman r	Confidence Interval	*p*
Temperature	0.07	0.02 to 0.11	0.001
pH	0.04	0.01 to 0.07	0.020
Free chlorine	−0.10	−0.12 to −0.06	<0.001
Combined chlorine	0.01	−0.02 to 0.04	0.590
Cyanuric acid	0.05	0.01 to 0.10	0.014
Bromine	−0.30	−0.40 to −0.19	<0.001
Turbidity	0.06	0.03 to 0.10	<0.001
Conductivity	−0.01	−0.07 to 0.04	0.652
Total dissolved solids	−0.01	−0.07 to 0.04	0.652
*Escherichia coli*	0.12	0.08 to 0.15	<0.001

## Data Availability

The original contributions presented in the study are included in the article; further inquiries can be directed to the corresponding author.
